# Self-supervised semantic segmentation of retinal pigment epithelium cells in flatmount fluorescent microscopy images

**DOI:** 10.1093/bioinformatics/btad191

**Published:** 2023-04-17

**Authors:** Hanyi Yu, Fusheng Wang, George Teodoro, Fan Chen, Xiaoyuan Guo, John M Nickerson, Jun Kong

**Affiliations:** Department of Computer Science, Emory University, Atlanta, GA 30322, USA; Department of Computer Science, Stony Brook University, Stony Brook, NY 11794, USA; Department of Computer Science, Federal University of Minas Gerais, Belo Horizonte 31270, Brazil; Huangpu Branch, Guangzhou Urban Planning & Design Survey Research Institute, Guangzhou 510060, China; Department of Computer Science, Emory University, Atlanta, GA 30322, USA; Department of Ophthalmology, Emory University, Atlanta, GA 30322, USA; Department of Computer Science, Emory University, Atlanta, GA 30322, USA; Department of Mathematics and Statistics, Georgia State University, Atlanta, GA 30303, USA

## Abstract

**Motivation:**

Morphological analyses with flatmount fluorescent images are essential to retinal pigment epithelial (RPE) aging studies and thus require accurate RPE cell segmentation. Although rapid technology advances in deep learning semantic segmentation have achieved great success in many biomedical research, the performance of these supervised learning methods for RPE cell segmentation is still limited by inadequate training data with high-quality annotations.

**Results:**

To address this problem, we develop a Self-Supervised Semantic Segmentation (S^4^) method that utilizes a self-supervised learning strategy to train a semantic segmentation network with an encoder–decoder architecture. We employ a reconstruction and a pairwise representation loss to make the encoder extract structural information, while we create a morphology loss to produce the segmentation map. In addition, we develop a novel image augmentation algorithm (AugCut) to produce multiple views for self-supervised learning and enhance the network training performance. To validate the efficacy of our method, we applied our developed S^4^ method for RPE cell segmentation to a large set of flatmount fluorescent microscopy images, we compare our developed method for RPE cell segmentation with other state-of-the-art deep learning approaches. Compared with other state-of-the-art deep learning approaches, our method demonstrates better performance in both qualitative and quantitative evaluations, suggesting its promising potential to support large-scale cell morphological analyses in RPE aging investigations.

**Availability and implementation:**

The codes and the documentation are available at: https://github.com/jkonglab/S4_RPE.

## 1 Introduction

**Figure 1:**
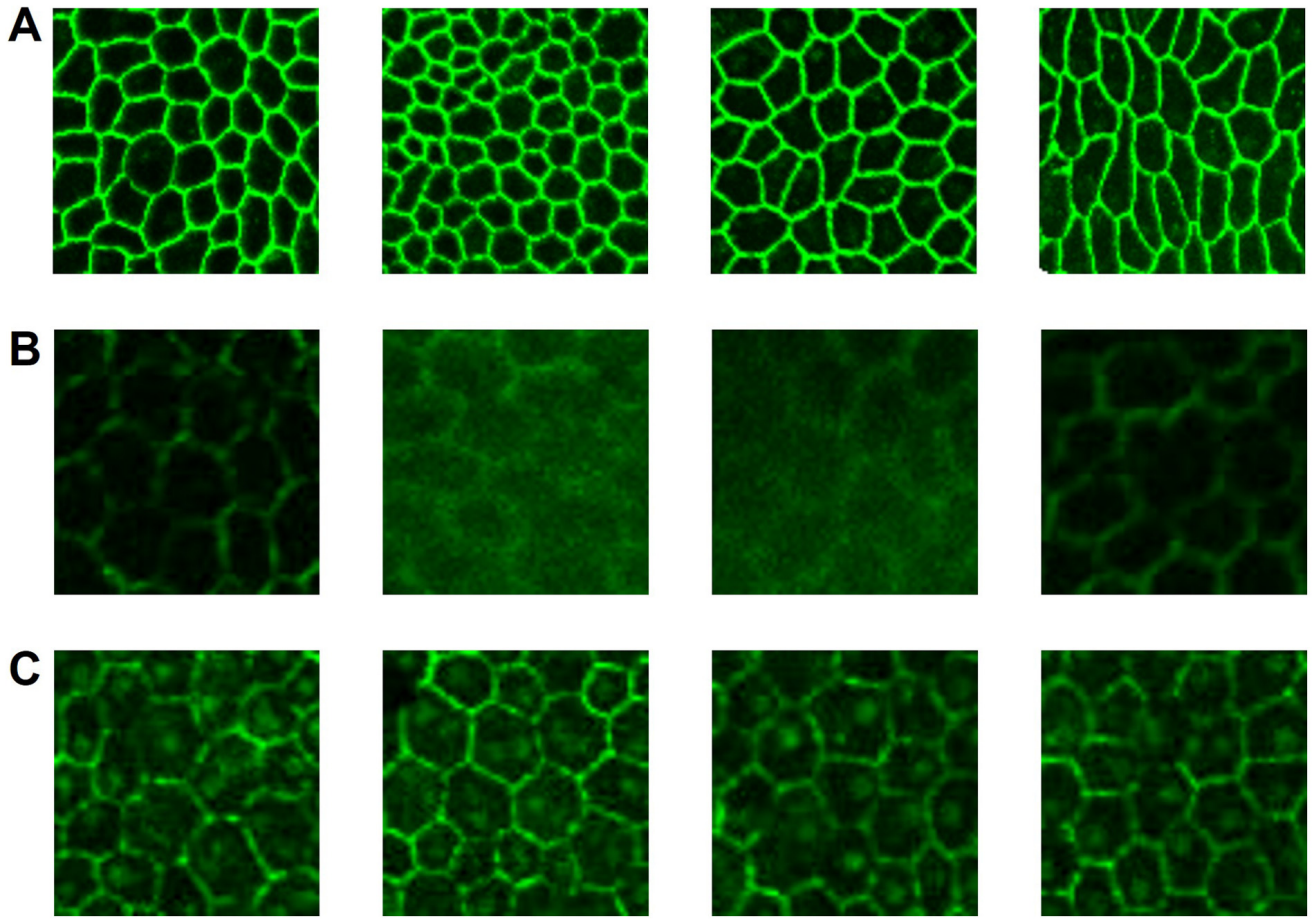
Representative RPE flatmount fluorescent microscopy image regions. (A) RPE cells in normal regions often have cell borders with high contrast. (B) RPE cells in damaged regions present weak or missing cell borders with a partial or complete cell structure loss. (C) RPE cell nuclei in damaged regions may be artifactually labeled, making it more challenging for accurate RPE cell segmentation

The age-related macular degeneration (AMD) is the primary cause of the central vision loss and legal blindness in the world (Ambati et al. 2003). The pigmented cell layer between the choroid and the neurosensory retina, known as the retinal pigment epithelium (RPE), plays an important role in the AMD pathogenesis. RPE primary functions include secreting immunosuppressive factors, maintaining photoreceptor excitability, and transporting nutrients (Strauss 2005). Therefore, RPE aging often results in secondary photoreceptor degradation, which ultimately leads to irreversible vision loss in turn. RPE cell morphological characteristics, such as area, perimeter, and aspect ratio, have previously been proven as good indicators of the cell pathophysiologic status and the degree of RPE aging (Ach et al. 2014; Bhatia et al. 2016; Kim et al. 2021). RPE flatmount fluorescent microscopy images have been widely used to calculate RPE cell morphological features. Before cell morphological features can be computed, cell borders have to be detected from flatmount images. Some typical RPE cell examples in flatmount images are illustrated in Fig. 1A. However, the flatmount image acquisition procedure inevitably produces damaged image regions where the RPE cells are often degraded by noise. Some RPE cells with blurred or missing cell borders from damaged image regions are presented in Fig. 1B. To facilitate an accurate cell border recovery, we would like to suppress the nucleus contrast in the resulting flatmount images. In practice, the imperfect tissue preparation, fluorescent protein labeling, and imaging processing often produce RPE cells with highlighted nuclei in practice (Fig. 1C). As the manual annotation process for such degraded image regions is time-consuming and suffers from a large inter- and intravariability, annotated training data are rarely adequate to support supervised learning methods. In our previous work (Yu et al. 2022), we developed a deep neural network for RPE cell border detection that was trained with a semisupervised learning strategy. With this approach, the training set was further enriched by unlabeled data. The trained model, thus, benefited from such an increase in training data scale and sample diversity. However, its performance is still subject to the limited labeled data in the training dataset.

To address this problem, we present in this paper a novel **S**elf-**S**upervised **S**emantic **S**egmentation (S^4^) method that leverages a self-supervised learning strategy. Note that this RPE cell segmentation method only requires unlabeled flatmount image data to train deep neural networks. Specifically, we utilize the reconstruction and pairwise representation loss to train an encoder-decoder architecture that recovers degraded image regions. For the pairwise representation learning, we develop a new image augmentation algorithm (AugCut) to generate correlated views of input images. Moreover, we formulate a novel morphology loss that incentivizes the network to generate binary outputs with closed cell borders. The developed approach is compared with the state-of-the-art deep learning approaches and demonstrates its superior performance for RPE cell segmentation with flatmount microscopy images. Ablation experiments present the necessity and efficacy of our training strategy design. With extensive tests and rigorous comparisons, the experimental performance of the proposed deep learning method suggests its promising potential to support large-scale cell morphological analyses for RPE aging studies.

## 2 Materials and methods

### 2.1 Literature review

Commonly used in biomedical research, semantic segmentation assigns a class label to each pixel in an image. Of all methods, a large number of deep learning approaches have been used for image semantic segmentation (Haque and Neubert 2020; Asgari Taghanaki et al. 2021). For example, fully convolutional network (FCN) creates segmentation maps by replacing fully connected layers in classification neural networks with deconvolution layers (Long et al. 2015). Built upon FCN, DeepLab successfully improves the performance by such new components as atrous convolution, conditional random field, and spatial pyramid pooling (Chen et al. 2018). Additionally, UNet adopts a symmetric encoder–decoder structure with skip connections between the encoder and the decoder at each resolution level (Ronneberger et al. 2015). Due to its excellent performance, a large number of variants of UNet have been proposed for biomedical image segmentation tasks (Li et al. 2018; Wang et al. 2018; Ibtehaz and Rahman 2020).

Self-supervised learning is a collection of unsupervised learning methods that extract information from unlabeled data and build good representations to facilitate downstream tasks. By the training sample organization strategy, self-supervised learning includes two broad categories: self-prediction and pairwise representation learning (Li et al. 2022). Typical self-prediction methods have a bottleneck architecture that encodes a high-dimensional input corrupted with artificial noise into a latent low-dimensional code and reconstructs the input signal from the latent representation (Kingma and Welling 2014; Higgins et al. 2017; van den Oord et al. 2017). Additionally, some other self-prediction methods are designed to drop a part of the input signal and recover the missing input component for the predicted output in training (Devlin et al. 2018; Bao et al. 2022; He et al. 2022). Another set of self-prediction methods transforms input data in a way that preserves the original input information with a desired innate logic (e.g. rotation or jigsaw). In this way, they provide supervision without task-specific labels (Gidaris et al. 2018; Jenni and Favaro 2018). In contrast, pairwise representation learning is a group of unsupervised learning methods where inputs from different branches are encoded as latent representations for loss calculation. In principle, the pairwise representation learning associates representations derived from related inputs and disassociates unrelated input representations. Following this idea, some studies adopt a contrastive loss function called InfoNCE (Oord et al. 2018) to reduce the representation similarity when augmented data with different semantics (i.e. negative pairs) are compared (Zhuang et al. 2019; He et al. 2020; Tian et al. 2020). In such study implementation, latent representations of previous samples are often stored in a dynamically updated memory bank and are used as negative representations for training. In addition, other studies [e.g. BYOL (Grill et al. 2020), SwAV (Caron et al. 2020), SimSiam (Chen and He 2021), and DINO (Caron et al. 2021)] generate multiple augmented views for the same input sample and train models to associate them with large similarity in the representation space.

Image augmentation is a set of techniques to increase the training dataset scale. It can significantly improve deep learning performance (Shorten and Khoshgoftaar 2019). Image augmentation is also a critical component in pairwise representation learning that creates multiple views with the same semantics but in different appearances. In addition to such image translations as random rotation, shifts, shear, and flips, the image augmentation method Mixup trains a neural network with pairwise convex combinations of samples and their labels (Zhang et al. 2018). In another study, CutMix improves the regional dropout with a new strategy where image patches are randomly cut and pasted across training images. The resulting ground truth label of each augmented image is computed by the proportions of image areas of different labels (Yun et al. 2019). Instead of mixing different samples, AugMix generates multiple augmented views for one sample and mixes these views by a convex combination (Hendrycks et al. 2020). RandConv uses a convolution operation with random parameters to create new views. The resulting convolutions serve as augmentation operators with similar global shapes but random local textures (Xu et al. 2021).

### 2.2 Deep neural network architecture

**Figure 2:**
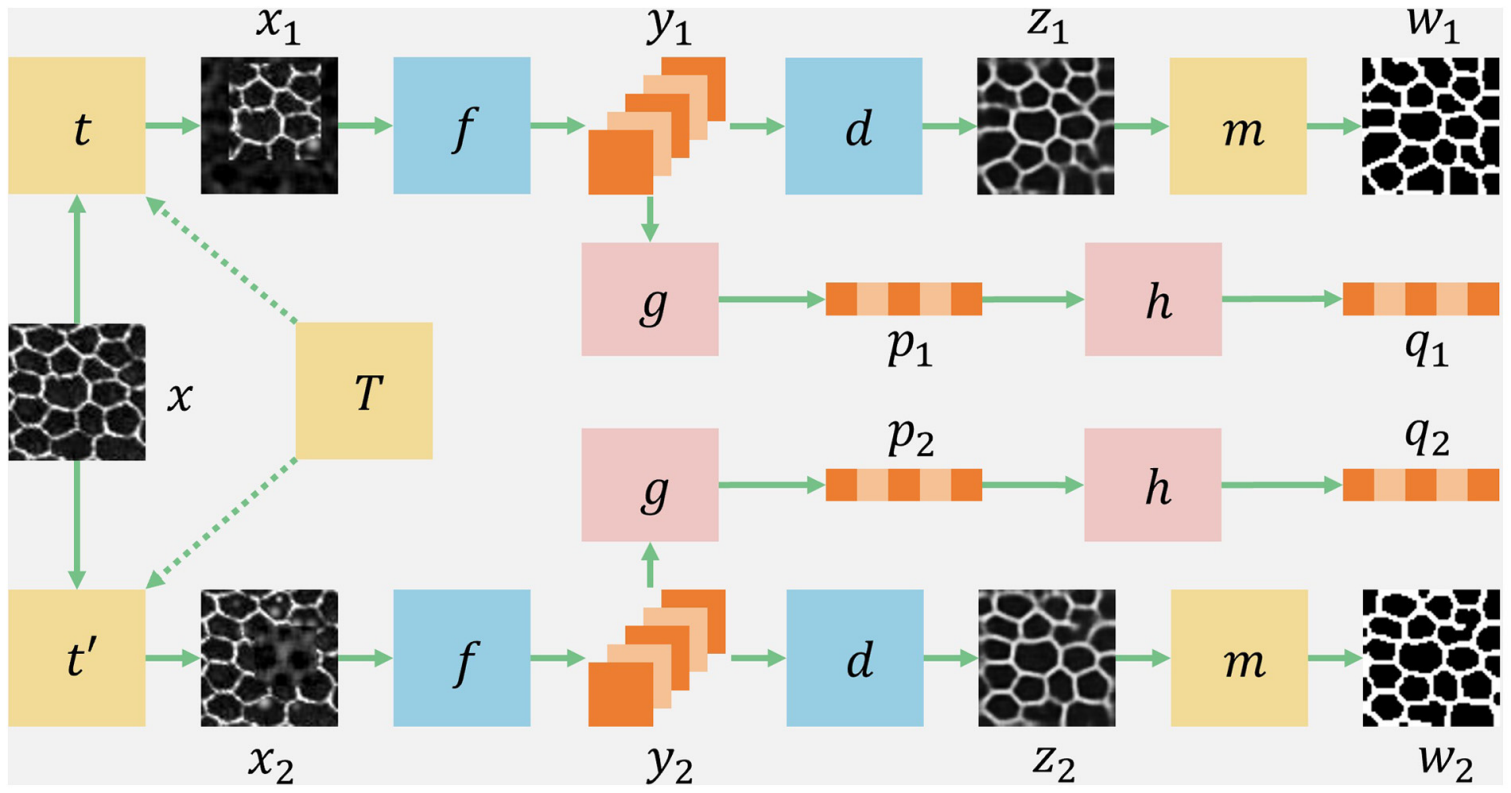
Overall schema of the developed self-supervised learning method S^4^. For each image input *x*, two different augmented views $x_{1}$ and $x_{2}$ are generated with operator *t* and $t^{\prime }$ randomly sampled from augmentation operator family *T*. Next, $x_{1}$ and $x_{2}$ are individually processed by the following convolutional and MLP layers for pairwise representation learning. The resulting latent feature vectors ($p_{1}$, $p_{2}$, $q_{1}$, and $q_{2}$) are used for pairwise representation loss. Additionally, $x_{1}$ and $x_{2}$ are processed by an encoder-decoder network and morphology operations with output images ($z_{1}$, $z_{2}$, $w_{1}$, and $w_{2}$) for reconstruction loss and morphological loss computation

An overview of our developed self-supervised learning method S^4^ is illustrated in Fig. 2. In the training stage, each input image *x*, is transformed into two related views (i.e. $x_{1}$ and $x_{2}$) by two image augmentation operators randomly sampled from the augmentation operator family *T*. Next, an encoder network *f* down-samples $x_{1}$ and $x_{2}$ into latent image representations (i.e. $y_{1}$ and $y_{2}$) that are further transformed into latent representation vectors (i.e. $p_{1}$ and $p_{2}$) by a multilayer perceptron (MLP) projection head *g*. We use another MLP prediction head *h* to match the mapped representation from one view to another latent representation vector [i.e. $p_{1}$ matching $q_{2}=h(p_{2})$, whereas $p_{2}$ matching $q_{1}=h(p_{1})$; Chen and He 2021]. The similarity between two views can be evaluated by the cosine similarity:


(1) $$D(q_{1},p_{2})=\frac{q_{1}\cdot p_{2}}{{\left\|q_{1}\right\|}_{2}{\left\|p_{2}\right\|}_{2}}$$

Prior studies have shown the necessity of using the stop-gradient operation, denoted as *sg*, in the pairwise learning (Grill et al. 2020; Chen and He 2021). For symmetry, the pairwise representation loss is defined as:


(2) $$L_{\text{PR}}=-\frac{1}{2}D\left(q_{1},sg\left(p_{2}\right)\right)-\frac{1}{2}D\left(q_{2},sg\left(p_{1}\right)\right).$$

As the defined pairwise representation loss guides the encoder *f* to extract structural information from impaired image regions, we also need loss functions to train the decoder network *d*. We denote the outputs of the decoder on two branches as $z_{1}=d(y_{1})$ and $z_{2}=d(y_{2})$, respectively. The performance of the decoder can be improved by minimizing the difference between input x and outputs $z_{1}$, $z_{2}$:


(3) $$L_{\text{Rec}\_i}=\frac{1}{2}{\left\|x-z_{1}\right\|}_{1}+\frac{1}{2}{\left\|x-z_{2}\right\|}_{1}.$$

In addition to the comparison between input and outputs, the reconstruction quality of the network can be evaluated by the difference between outputs $z_{1}$, $z_{2}$:


(4) $$L_{\text{Rec}\_o}={\left\|z_{1}-z_{2}\right\|}_{1}.$$

Combining loss term from Equations (3) and (4), we define the reconstruction loss as: where $\lambda_{1}$ is a weight factor ranging from 0 to 1.


(5) $$L_{\text{Rec}}=\lambda_{1}L_{\text{Rec}\_i}+(1-\lambda_{1})L_{\text{Rec}\_o},$$

In the ideal situation, the reconstruction loss $L_{\text{Rec}}$ can be reduced to zero and outputs $z_{1}$, $z_{2}$ are exactly the same as input *x*. However, our goal is to train a network that can generate a binary segmentation output for each input image. Therefore, we utilize morphological transformations *m* to produce a binary map *w* for *z* and design a morphology loss as a function of the difference between *w* and *z*. By minimizing this difference, we can guide the network to generate binary segmentation maps. We define the morphology loss term as: where $w_{1}=m(z_{1})$ and $w_{2}=m(z_{2})$.


(6) $$L_{\text{Mor}}=\frac{1}{2}{\left\|w_{1}-z_{1}\right\|}_{1}+\frac{1}{2}{\left\|w_{2}-z_{2}\right\|}_{1},$$

**Figure 3:**
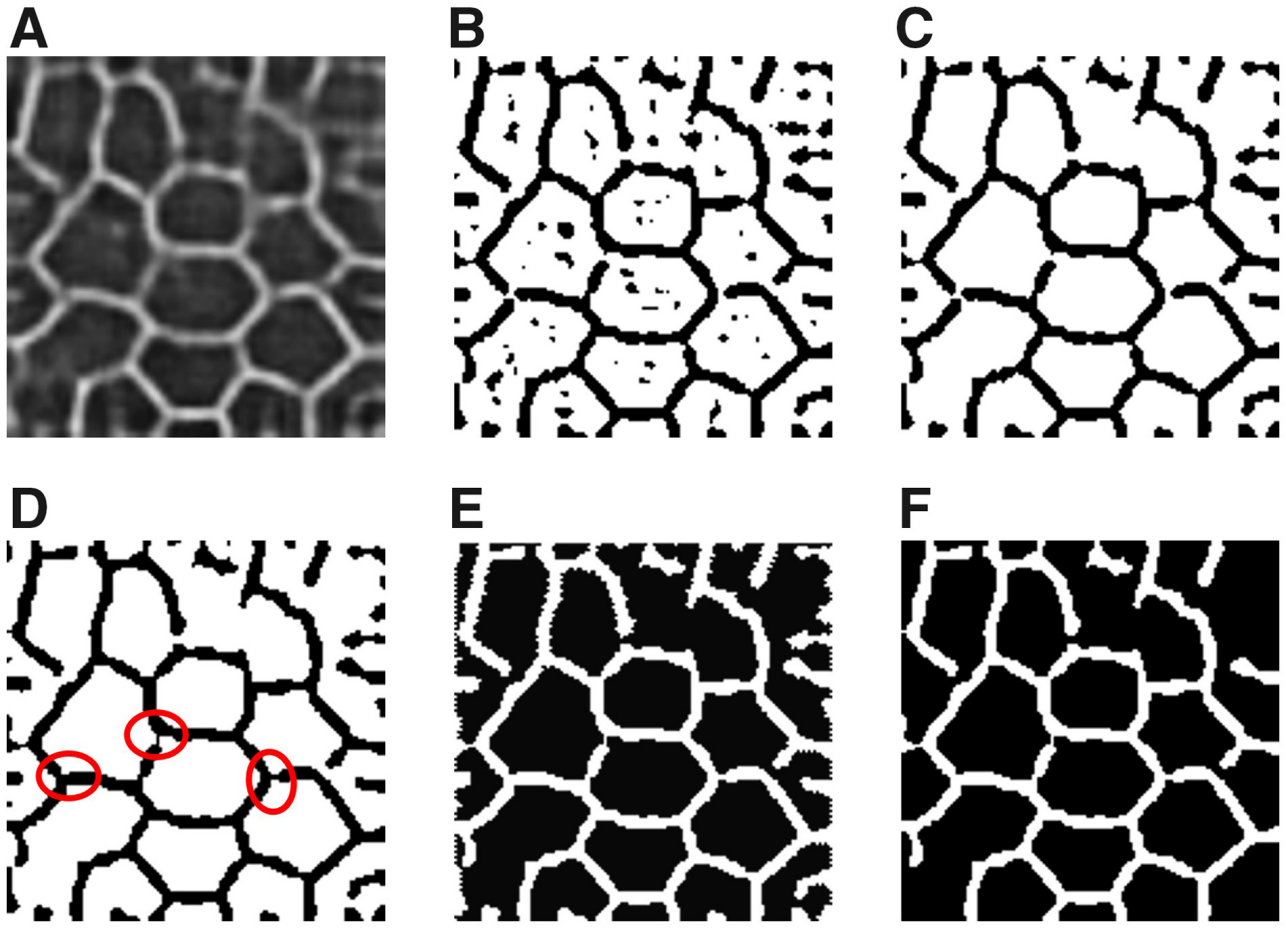
A typical example of the morphological transformation process. (A) A representative output image example z from the decoder; (B) The binarized result after adaptive thresholding; (C) Hole exclusion by filling external contours; (D) Border connections by the image opening operation (circles); (E) The reversed image; (F) Exclusions of small regions

We illustrate morphological transformation steps for our network training in Fig. 3. First, the output *z* from the decoder is binarized by the adaptive thresholding (Castleman 1996). Next, we remove the holes by extracting and filling the external contours. An image opening operation with a 3×3 structural element is used to connect borders. Finally, we reverse the image and remove small regions with area <10 pixels.

Finally, the overall loss function of our method is formulated as: where $\lambda_{2}$ and $\lambda_{3}$ are loss weight factors.


(7) $$L=L_{\text{PR}}+\lambda_{2}L_{\text{Rec}}+\lambda_{3}L_{\text{Mor}},$$

### 2.3 Image augmentation

**Figure 4:**
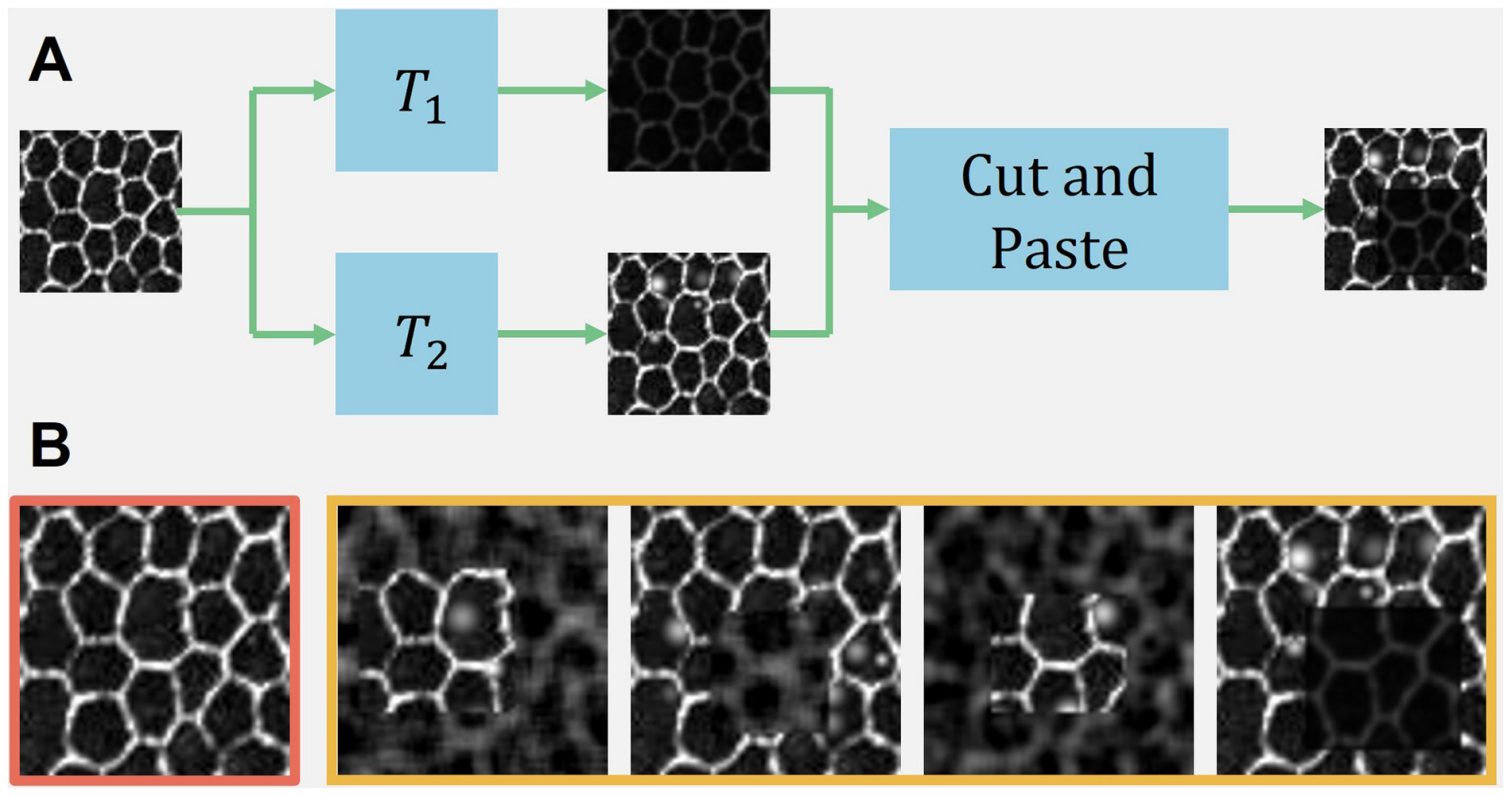
Developed image data augmentation algorithm AugCut. (A) Each input image is processed by two augmentation branches for brightness reduction, random image blur, and noise addition. With the resulting two output images, we randomly cut an image subregion from one and paste it to the other at the same image location. (B) We present a typical input image (left) and its augmented views (right)

Our developed augmentation algorithm AugCut consists of two augmentation branches (Fig. 4A). To emulate RPE cells in damaged tissue regions, we produce augmented images from training images with clear cell borders sampled from our dataset. On the top branch, input images are corrupted by $T_{1}$ with random Gaussian noise, Gaussian blur, and brightness reduction. This branch imitates the images of damaged tissue regions in Fig. 1B where cell borders are blurred or even missing. In contrast, Gaussian-distributed blobs are added to input images at random locations by $T_{2}$ on the bottom branch, mimicking RPE cells with highlighted nuclei in Fig. 1C. Similar to CutMix (Yun et al. 2019), we randomly cut an image subregion from the output of $T_{1}$ on the top branch and paste it to the output of $T_{2}$ at the same position. Thus, the augmentation result can be described as: where *R* is a randomly selected rectangle region. In our implementation, we swap $T_{1}$ and $T_{2}$ with a probability of 0.5 for symmetry. In Fig. 4B, we present a typical input image (in red) and its augmented views (in yellow) by different augmentation operators sampled from the same augmentation operator family *T*.


(8) $$T(x;i,j)=\{\begin{array}{cc} T_{1}(x;i,j) & (i,j)\in R\\ T_{2}(x;i,j) & (i,j)\notin R \end{array}.$$

### 2.4 Network training implementation

We implement our method S^4^ with the Python 3.10 and PyTorch 1.8.1 deep learning toolkit (Paszke et al. 2019) and execute programs on two NVIDIA Tesla K80 GPUs. The encoder *f* consists of an input layer, three down-sampling blocks using convolution layers with stride 2, and six residual blocks. The decoder *d* consists of three up-sampling blocks using deconvolution layers with stride 1/2 and an output layer. Balancing the tradeoff between computational efficiency and deep network efficacy, we adopt 64, 128, 256, and 512 filters from the highest to the lowest resolution level, respectively. MLP head *g* and *h* include two and one hidden layers with 512 nodes, respectively. The length of the output representation vector from *g* and *h* is 2,048 each.

During training, the loss function first guides the network to extract structural information and recover the input image. After this, the loss function guides the network to produce binary segmentation images with closed cell borders. To realize this two-stage training, we adopt a dynamic factor λ: where $t_{1}$ and $t_{2}$ are transition “time” cutoff values in the unit of epoch; *s* and *e* are the constant values before and after the transition. For factor $\lambda_{1}$, we have settings: $s=1,\;e=0.5,\;t_{1}=40,\;t_{2}=70$. For factor $\lambda_{2}$, we set it to constant 0.5. For factor $\lambda_{3}$, we have settings: $s=0,\;e=1,\;t_{1}=40,\;t_{2}=70$.


(9) $$\lambda (t)=\{\begin{array}{cc} s & t\leq t_{1}\\ s-\frac{s-e}{t_{2}-t_{1}}(t-t_{1}) & t_{1}<t<t_{2}\\ e & t\geq t_{2} \end{array},$$

## 3 Results

### 3.1 Dataset and evaluation metrics

In this study, the mouse RPE flatmount images are selected from our image database (Jiang et al. 2013; Boatright et al. 2015). As these RPE images have high image resolutions (around 4000 × 4000 pixels each), we divide each image into small image patches of 96 × 96 pixels for model training. Although the developed method S^4^ only requires unlabeled images with strong borders, the state-of-the-art methods in our comparison study require additional training data. Therefore, both labeled and unlabeled RPE cells are included in our training set. For the annotated set P, we have 155 patches with manually annotated ground truth. Regarding unlabeled RPE cells for training, we have two subsets. One high-quality image set X includes 653 patches and another low-quality Y includes 987 image patches. For the testing set, we include 43 258 RPE cells from 300 image patches.

We use multiple evaluation metrics for RPE cell segmentation evaluation. In image segmentation tasks, metrics derived from the confusion matrix are frequently utilized (Long et al. 2015; Ronneberger et al. 2015; Chen et al. 2018). In this work, we assign the positive class to border pixels and the negative class to pixels not on borders. Given these two classes, the confusion matrix has four values: TP (number of correctly classified pixels on borders), FP (number of incorrectly classified pixels not on borders), FN (number of incorrectly classified pixels on borders), and TN (number of correctly classified pixels not on borders). With these values, we compute Precision (Pre), Recall (Rec), Intersection-over-union (IOU), and Dice similarity coefficient (DSC) for method performance evaluation:


(10) $$\begin{array}{cc} \text{Pre}=\frac{\text{TP}}{\text{TP}+\text{FP}}, & \text{Rec}=\frac{\text{TP}}{\text{TP}+\text{FN}}\\ \text{IOU}=\frac{\text{TP}}{\text{TP}+\text{FP}+\text{FN}}, & \text{DSC}=\frac{\text{TP}\times 2}{\text{TP}\times 2+\text{FP}+\text{FN}} \end{array}.$$

As we aim to utilize segmentation results for down-stream morphological feature extraction, we introduce correct rate (CR) and weighted correct rate (WCR) as two additional metrics that assess the segmentation performance by RPE cell topology (Yu et al. 2022).

### 3.2 Model validation and performance comparison

We compare our developed method S^4^ with four supervised learning models [i.e. UNet (Ronneberger et al. 2015), DeepLab (Chen et al. 2018), MultiResUNet (Ibtehaz and Rahman, 2020), and Cellpose (Stringer et al. 2021)], and two semisupervised learning methods [i.e. UNet enhanced with CUT (Park et al. 2020) and MultiHeadGAN (Yu et al. 2022)]. UNet, DeepLab, and MultiResUNet have been widely applied to a large number of biomedical image segmentation tasks (Zhou et al. 2021). Cellpose adopts a pretrained UNet model to predict gradient vector fields and segment cells by gradient tracking. Two semisupervised approaches using the pairwise learning mechanism have been developed to use unlabeled data for model training (Yu et al. 2022). For method comparison, we train supervised learning approaches with the labeled training set P and semisupervised learning approaches with training sets P, X, and Y.

**Figure 5:**
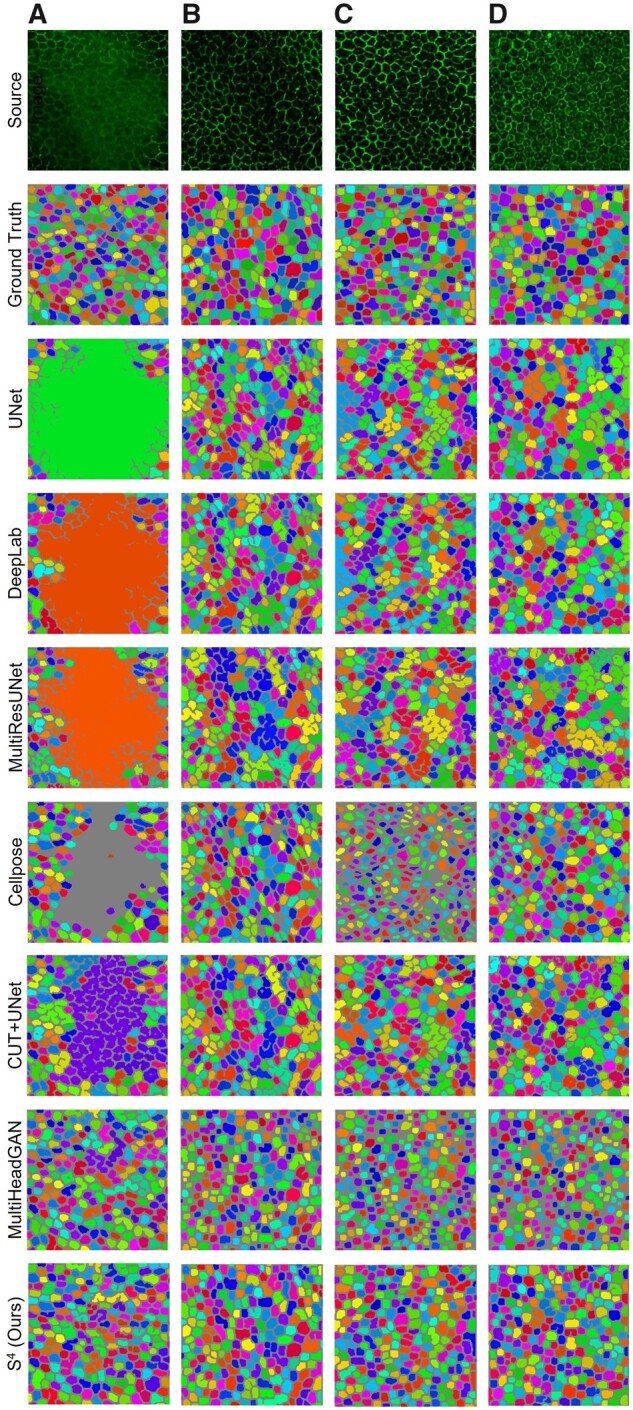
Qualitative comparison of deep learning approaches for RPE cell segmentation with flatmount microscopy images. Four typical impaired image regions are shown in columns (A–D) with rows for ground truth and corresponding segmentation results of UNet, DeepLab, MultiResUNet, Cellpose, CUT + UNet, MultiHeadGAN, and our S^4^, respectively. Column (A) demonstrates the case that the whole region is severely blurred, while columns (B and C) present cases where cell borders are partially missing. Column (D) presents the case where RPE cells contain highlighted nuclei

We present and compare typical RPE cell segmentation results of these models in Fig. 5. By visual comparisons, the results from supervised learning approaches except for Cellpose have large undersegmented regions. Semisupervised learning approaches can effectively mitigate under-segmentation, while our developed method S^4^ achieves the best overall performance. Note that the performance of Cellpose is the second best as it is trained with a large amount of labeled data. However, Cellpose fails to produce reasonable RPE cell segmentation results when the gradient vectors in cells do not converge.

We also quantitatively evaluate and compare segmentation results from different models with multiple metrics (i.e. Pre, Rec, IOU, DSC, CR, and WCR). The quantitative comparison results are shown in Table 1. Our developed method S^4^ achieves the best performance by Pre (85.0%), IOU (76.8%), DSC (86.7%), CR (88.9%), and WCR (89.9%), respectively. Additionally, quantitative evaluation results are plotted in Supplementary Fig. S1 to present the statistical difference between our developed method and others. By Pre, IOU, and DSC, our method S^4^ is significantly better than all other approaches. By CR and WCR, S^4^ is significantly better than all other approaches except Cellpose. Although MultiHeadGAN achieves the best performance by Rec, the difference between S^4^ and MultiHeadGAN is negligible (i.e. 1.7% with *P *=* *.08).

**Table 1.** Quantitative performance comparison across our developed method S^4^ and deep learning methods by multiple evaluation metrics.^a^

Method	Pre	Rec	IOU	DSC	CR	WCR
Supervised						
UNet	74.4	67.1	54.5	70.0	53.1	57.0
Deeplab	74.4	68.6	55.5	71.0	62.6	66.3
MultiResUNet	76.7	79.9	64.2	78.0	62.3	65.2
Cellpose	68.2	89.0	62.8	76.7	88.8	89.2
Semisupervised						
CUT + UNet	78.6	82.7	67.4	80.5	72.2	75.3
MultiHeadGAN	61.2	**90.1**	57.5	72.9	84.3	85.6
Self-supervised						
S^4^ (Ours)	**85.0**	88.4	**76.8**	**86.7**	**88.9**	**89.9**

a All values are in percentage (%) and the best value in each column is highlighted in bold.

### 3.3 Ablation study

We present a set of ablation experiments to demonstrate the efficacy of (i) loss term combinations, (ii) pairwise learning strategies, (iii) data augmentation methods, and (iv) dynamic loss term weight factors for training.

To study the contribution of individual training losses in our developed S^4^ method, we carry out ablation experiments with different loss term combinations (Table 2). As the reconstruction loss $L_{\text{Rec}}$ is necessary for training, we keep this loss term and only test with different combinations of $L_{\text{PR}}$ and $L_{\text{Mor}}$. Note that the resulting output image is in grayscale when $L_{\text{PR}}$ is not used. For fair comparisons, we use the same morphological transformations (Section 3.1) to binarize the grayscale output in such a case. In Table 2, when individually combined with $L_{\text{Rec}}$, the loss term $L_{\text{Mor}}$ presents better performance than $L_{\text{PR}}$ by Rec (+25.6%), IOU (+18.2%), DSC (+11.1%), CR (+110.1%), and WCR (+92.6%), respectively. Additionally, the combination of all three loss terms used in our developed S^4^ achieves the best performance. This suggests that the pairwise representation loss and the morphology loss are complementary to the reconstruction loss term, contributing to an enhanced method performance. Supplementary Fig. S2 presents the statistical difference across different training loss combinations by the evaluation metrics.

**Table 2.** Quantitative performance comparison across different training loss combinations by multiple evaluation metrics.^a^

Loss			Pre	Rec	IOU	DSC	CR	WCR
$L_{\text{Rec}}$	$L_{\text{PR}}$	$L_{\text{Mor}}$						
**✓**	**✗**	**✗**	79.5	61.8	53.6	69.0	38.6	40.5
**✓**	**✓**	**✗**	83.6	65.3	58.1	73.1	40.4	43.5
**✓**	**✗**	**✓**	80.7	82.0	68.7	81.2	84.9	83.8
**✓**	**✓**	**✓**	**85.0**	**88.4**	**76.8**	**86.7**	**88.9**	**89.9**

a Included and excluded terms are checked by “✓” and “✗,” respectively. All values are in percentage (%) and the best value in each column is highlighted in bold.

We further test the impact of different self-supervised learning strategies on segmentation performance. In our developed method, we adopt SimSiam (Chen and He 2021) to compute the pairwise representation loss with augmented view $x_{1}$ and $x_{2}$. In the ablation study, we include an additional pairwise representation loss term $L_{\text{PR}}^{\prime }$ related to outputs (i.e. $z_{1}$ and $z_{2}$). Thus, we process $z_{1}$ with encoder *f*, MLP head *g*, and *h* in sequence and obtain $p_{1}^{\prime }=g(f(z_{1}))$ and $q_{1}^{\prime }=h(p_{1}^{\prime })$. Similarly, we compute $p_{2}^{\prime }$ and $q_{2}^{\prime }$ with $z_{2}$. The resulting overall pairwise representation loss is related to both input and outputs, and formulated as $\frac{1}{2}L_{\text{PR}}+\frac{1}{2}L_{\text{PR}}^{\prime }$ with $L_{\text{PR}}^{\prime }$ defined as:


(11) $$L_{\text{PR}}^{\prime }=-\frac{1}{2}D\left(q_{1}^{\prime },sg\left(p_{2}^{\prime }\right)\right)-\frac{1}{2}D\left(q_{2}^{\prime },sg\left(p_{1}^{\prime }\right)\right).$$

Similar to the ablation studies with SimSiam, we formulate the pairwise representation loss with another state-of-the-art strategy BYOL (Grill et al. 2020) with only inputs, and both inputs and outputs. The experimental results are presented in Table 3. The difference between BYOL and SimSiam is not significant by Pre (*P *=* *.86), Rec (*P *=* *.89), IOU (*P *=* *.99), DSC (*P *=* *.98), CR (*P *=* *.75), or WCR (*P *=* *.78). As BYOL includes an online and a target network, it is more time-consuming and takes more memory for model training than SimSiam. In our implementation, we choose the learning strategy by SimSiam. Interestingly, we notice that the addition of the pairwise representation loss by outputs can impair the model performance. Supplementary Fig. S3 presents the statistical difference across different pairwise representation learning strategies by the evaluation metrics.

**Table 3.** Quantitative performance comparison across different pairwise representation learning strategies by multiple evaluation metrics.^a^

Learning Strategy	Loss from		Pre	Rec	IOU	DSC	CR	WCR
	I	O						
BYOL	**✓**	**✓**	84.4	84.4	73.2	84.2	81.4	84.3
	**✓**	**✗**	84.8	**88.6**	**76.8**	86.6	**89.2**	**90.2**
SimSiam	**✓**	**✓**	**86.9**	84.0	75.0	85.4	83.6	85.6
	**✓**	**✗**	85.0	88.4	**76.8**	**86.7**	88.9	89.9

a Included and excluded terms are checked by “✓” and “✗,” respectively. I, input views $x_{1},x_{2}$; O, outputs $z_{1},z_{2}$. All values are in percentage (%) and the best value in each column is highlighted in bold.

We present the effectiveness of the developed image augmentation algorithm AugCut by experimental results in Table 4. For comparison, we present the testing performance associated with $T_{1}$ and $T_{2}$ individually. Additionally, we show an enhanced testing performance with input views randomly augmented by either $T_{1}$ or $T_{2}$. Of all augmentation strategies for comparison, AugCut mixing results by $T_{1}$ and $T_{2}$ presents the best performance. Supplementary Fig. S4 presents the statistical difference across different image augmentation strategies by the evaluation metrics.

**Table 4.** Quantitative performance comparison among different image augmentation strategies by multiple evaluation metrics.^a^

Augmentation strategy	Pre	Rec	IOU	DSC	CR	WCR
$T_{1}$	**86.9**	84.0	75.0	85.4	83.6	85.7
$T_{2}$	80.3	72.8	61.9	76.1	53.4	56.0
Random	83.7	87.3	75.0	85.4	84.5	85.2
AugCut	85.0	**88.4**	**76.8**	**86.7**	**88.9**	**89.9**

a All values are in percentage (%) and the best value in each column is highlighted in bold.

We investigate the optimal transition cutoff time settings (i.e. $t_{1}$ and $t_{2}$) for loss weight factor $\lambda_{1}$ and $\lambda_{3}$ (Table 5). Our study results suggest the strategy with fixed weight factors (i.e. $t_{1}=t_{2}=0$) can successfully train a network with an acceptable overall performance. However, such a strategy is very sensitive to the network parameter initialization and often results in a degenerated network with unmeaningful outputs. Next, we gradually increase the starting (i.e. $t_{1}$) and ending time (i.e. $t_{2}$) for the transition. We define the stage before the transition as pretraining. Supplementary Fig. S5 suggests the network tends to generate outputs with blurred cell edges when the pretraining stage epoch is small. This makes it difficult for the morphological transformation process to recover cell borders. When the pretraining stage epoch is unduly large, the network tends to generate outputs similar to the original input image. This makes it hard for the morphological transformation process to produce continuous cell borders. In our work, we find that 40 is a proper epoch number for the pretraining stage by the morphology loss (Supplementary Fig. S6) and confirm it with both quantitative and qualitative experimental results. Supplementary Fig. S7 presents the statistical difference across different transition time cutoff values by the evaluation metrics.

**Table 5.** Quantitative performance comparison with different transition time cutoff values in the unit of epoch for weight factor $\lambda_{1}$ and $\lambda_{3}$.^a^

$t_{1}$	$t_{2}$	Pre	Rec	IOU	DSC	CR	WCR
0	0	**86.0**	78.4	69.8	81.9	83.5	85.1
0	30	/	/	/	/	/	/
20	50	77.7	73.5	60.9	75.4	61.9	65.1
40	70	85.0	**88.4**	**76.8**	**86.7**	**88.9**	**89.9**
60	90	83.2	80.9	69.9	82.0	80.9	83.1
80	110	81.4	78.4	66.7	79.7	80.1	81.7

a All evaluation metric values are in percentage (%) and the best value in each column is highlighted in bold.

**Figure 6:**
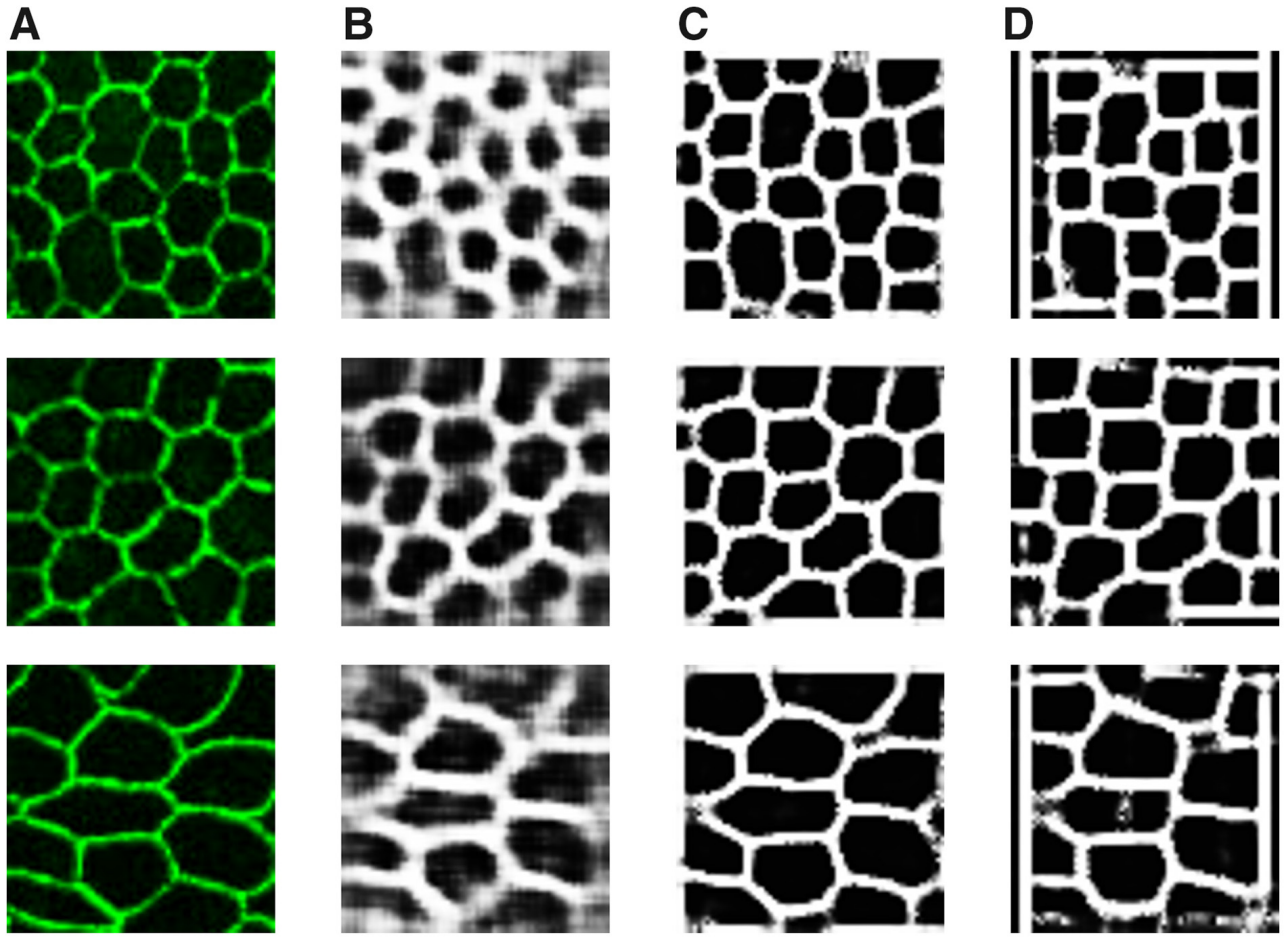
Typical output examples of networks trained with different stabilized values for weight factor $\lambda_{1}$. (A) Three typical input images; (B–D) Outputs associated with the stabilized value e=0.3,0.5,0.7, respectively

We also study the impact of different stabilized values (i.e. *e*) for the weight factor $\lambda_{1}$ on the method performance. We present typical segmentation examples with different *e* in Fig. 6. When *e* takes a large value, the loss term $L_{{\text{Rec}}_{i}}$ between input x and outputs (i.e. $z_{1}$ and $z_{2}$) imposes a strong constraint on outputs and prevents them from being binary. As a result, some cell borders in the outputs are still in grayscale. In contrast, a small value for *e* tends to make cell borders deviate from true cell border structures in the input images. By the ablation study, we make e=0.5 as this stabilized value selection can produce promising model performance by all evaluation metrics (Table 6). Supplementary Fig. S8 presents the statistical difference across different stabilized values by the evaluation metrics.

**Table 6.** Quantitative performance comparison among different stabilized values for weight factor $\lambda_{1}$ by multiple evaluation metrics.^a^

Stabilized value *e*	Pre	Rec	IOU	DSC	CR	WCR
0.3	75.9	76.4	61.6	76.0	78.3	79.8
0.5	**85.0**	**88.4**	**76.8**	**86.7**	**88.9**	**89.9**
0.7	74.3	81.2	63.5	77.4	83.1	85.5

a All values are in percentage (%) and the best value in each column is highlighted in bold.

## 4 Discussion

A precise and complete understanding of the RPE cell morphology is the key to improve our comprehension of RPE physiology and aging (Chrenek et al. 2012; Jiang et al. 2013; Kim et al. 2021). In turn, an accurate segmentation is a prerequisite for the morphological characterization. Due to lack of appropriate computational tools, RPE cell segmentation often depends on manual annotations, a process suffering from a large inter- and intravariability. Such a human annotation process is also too time-consuming to produce a sufficiently large number of annotated RPE cells in damaged tissue image regions for deep learning training. Thus, the human-annotated dataset is very limited. There are numerous training image patches with impaired RPE cell patterns not included in the training set, leading to the unsatisfying supervised learning performance. Such supervised learning limitations motivate us to leverage unlabeled image data on a much larger scale and develop a novel method (S^4^) for segmenting RPE cells in flatmount fluorescent microscopy images in this study.

Our developed method takes a self-supervised learning strategy and enables deep neural networks to learn from unlabeled image data, resulting in a more generalized feature extraction ability and learning outcome. Our idea is to produce synthetic damaged image regions by applying image augmentations to good quality image patches and train a network to recover good quality images by a reconstruction loss. To enhance our model performance, we combine this component with two MLP heads that extract representation vectors for the pairwise learning during the training. Our ablation study results in Table 2 manifest that our model benefits from this combination strategy by all included performance metrics. However, this loss combination can only produce grayscale images. To achieve RPE cell segmentation results in binary, we next design a RPE cell morphology loss that compares decoder outputs *z* with their binary results *w* after the designed morphological transformations. With the addition of the morphology loss, the network generates decoder outputs approaching binarized segmentation maps. In practice, we adopt a dynamic strategy to adjust the composition of loss terms as a function of the training epoch. At the beginning of the training stage, the network is trained with pairwise representation loss $L_{\text{PR}}$ and reconstruction loss $L_{\text{Rec}\_i}$ between input *x* and outputs (i.e. $z_{1}$ and $z_{2}$). After the training performance becomes stable, we decrease the weight of $L_{\text{Rec}\_i}$ to reduce the input-output similarity constraint. Concurrently, we increase the weight of reconstruction loss $L_{\text{Rec}\_o}$ to support the supervision by semantic information. Meanwhile, we increase the weight of morphology loss $L_{\text{Mor}}$ and gradually force the model to produce binary segmentation outputs.

In the ablation study, we present the superiority of our method design by experimental results of multiple variants of our method. Specifically, we observe that the addition of pairwise representation loss PR from outputs decreases the segmentation performance in Table 3. The core task for the encoder *f* is to extract structural information from damaged RPE image regions. However, the loss term $L_{\text{PR}}^{\prime }$ makes use of the encoder *f* to extract information from the binary output *z*, resulting in a performance decrease.

Data augmentation results in Table 4 suggest that the augmentation with $T_{1}$ performs better than the augmentation with $T_{2}$ in general. Recall that $T_{1}$ augments weak cell border cases, whereas $T_{2}$ produces cells with strong nuclei. As missing cell borders in the segmentation results can significantly alter the RPE cell topology, these cells have a higher impact on the method performance. Additionally, as both weak RPE cell borders and noisy tissue regions with highlighted nuclei are augmented in the corrupted image views by AugCut, it creates a rich data diversity, contributing to its best performance.

For further performance improvement, we plan to leverage “network engineering” techniques in future research. For example, attention layers are well known for making networks focus on informative features (Woo et al. 2018; Hu 2019; Niu et al. 2021). We will add them to our network to help extract RPE cell borders. We will also deploy transformer-based networks to replace convolutional layers in our study due to their promising performance in numerous medical image segmentation applications (Shamshad et al. 2022). Finally, we plan to generalize our approach and support more cell morphology related biomedical investigations. As the morphological transformations used in this work are customized to RPE cell analysis, they may require further changes to better support other cell related analyses.

## 5 Conclusion

We develop a S^4^ method that utilizes an unsupervised learning strategy to train a semantic segmentation deep learning network for RPE cell segmentation with low-quality flatmount fluorescent microscopy images. The developed method trains a deep neural network with unlabeled images. Specifically, a reconstruction loss by the encoder–decoder component and a pairwise representation loss by auxiliary MLP heads are used to extract structural information of cell borders. Additionally, a morphological loss guides the network to output binary segmentation results. The performance of the model is qualitatively and quantitatively evaluated and compared with other state-of-the-art deep learning approaches for cell analysis. Our experimental results suggest the superiority of our developed method, which is promising for analyses of RPE cell morphology. S^4^ will be useful in studies of the aging and pathology of RPE cells.

## Supplementary Material

btad191_Supplementary_Data
