# Supplementary material for: Self-supervised semantic segmentation of retinal pigment epithelium cells in flatmount fluorescent microscopy images
Source: Bioinformatics. 2023 Apr 17;39(4):btad191. doi: 10.1093/bioinformatics/btad191 (PMC10139776; doi:10.1093/bioinformatics/btad191)
Supplement: btad191_Supplementary_Data [file btad191_supplementary_data.pdf]

Self-supervised semantic segmentation of retinal pigment epithelium cells in flatmount fluorescent microscopy images

HANYI YU [1], FUSHENG WANG [2], GEORGE TEODORO [3], FAN CHEN [4], XIAOYUAN GUO [1], JOHN M. NICKERSON [5] AND JUN KONG [1,6,*]

[1] Department of Computer Science, Emory University, Atlanta, 30322, USA and [2] Department of Computer Science, Stony Brook University, Stony Brook, 11794, USA and [3] Department of Computer Science, Federal University of Minas Gerais, Belo Horizonte, 31270, Brazil and [4] Guangzhou Urban Planning and Design Survey Research Institute, Guangdong, 510060, China and [5] Department of Ophthalmology, Emory University, Atlanta, 30322, USA and [6] Department of Mathematics and Statistics, Georgia State University, Atlanta, 30303, USA.

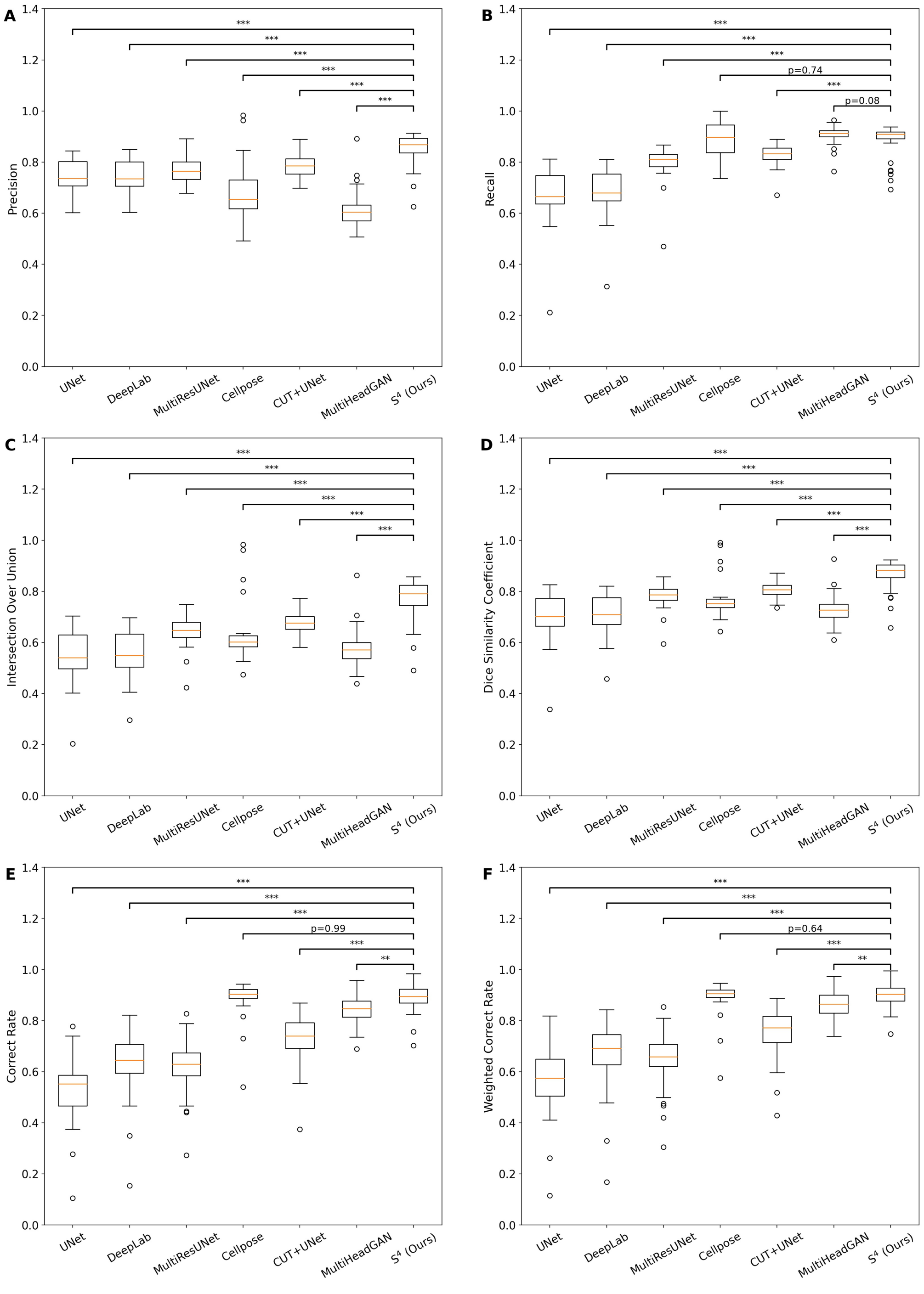

Fig. S1. Quantitative comparison of deep learning approaches for RPE cell segmentation. The RPE cell segmentation performance of deep learning models is evaluated and compared by (A) Precision, (B) Recall, (C) Intersection Over Union, (D) Dice Similarity Coefficient, (E) Correct Rate, and (F) Weighted Correct Rate. Paired sample t-tests between the developed S^4 and other six state-of-the-art methods suggest a statistically significant performance difference. The notations for *, **, and *** represent a *p*-value less than 0.05, 0.005, and 0.0005, respectively.

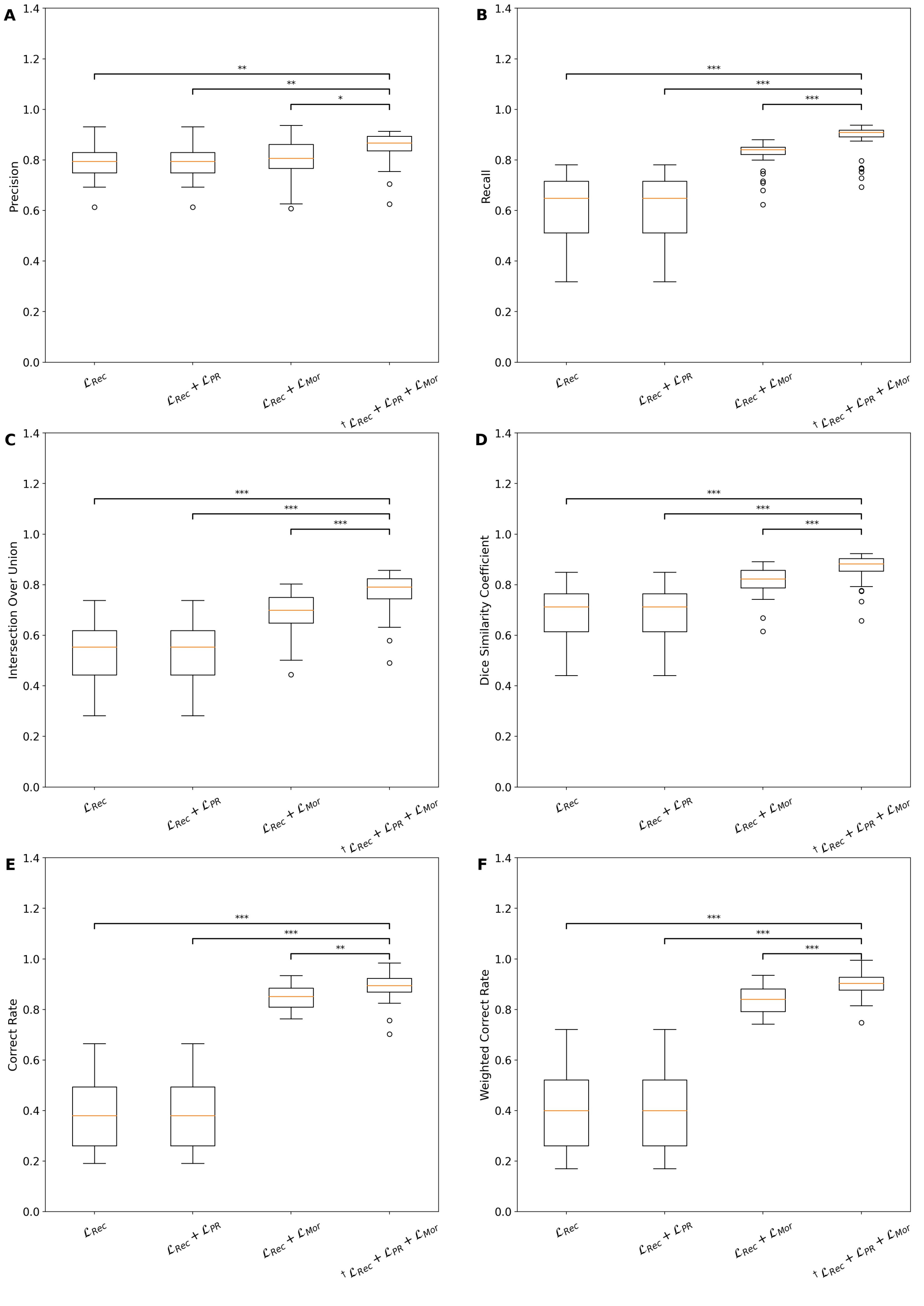

Fig. S2. Quantitative performance comparison across different training loss combinations by multiple evaluation metrics. We compare our method segmentation performance associated with different training loss combinations by (A) Precision, (B) Recall, (C) Intersection Over Union, (D) Dice Similarity Coefficient, (E) Correct Rate, and (F) Weighted Correct Rate. The marker † denotes the specific loss combination used in S^4. The notations for *, **, and *** represent a p-value less than 0.05, 0.005, and 0.0005, respectively.

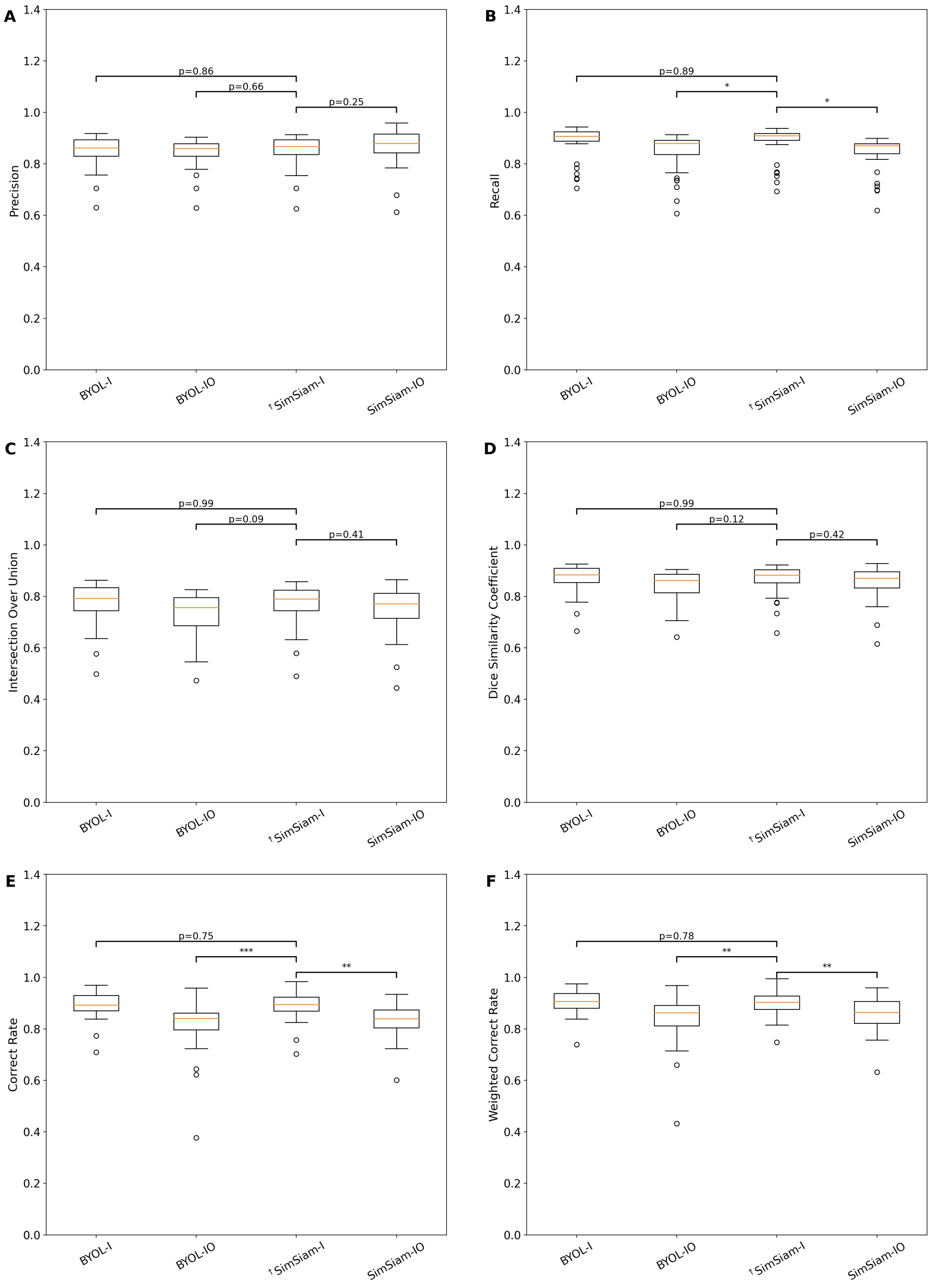

Fig. S3. Quantitative performance comparison across different pairwise representation learning strategies by multiple evaluation metrics. Four strategies for comparison are BYOL-I, BYOL-IO, SimSiam-I, and SimSiam-IO. BYOL and SimSiam are two different pairwise representation learning methods, with suffix ’-I’ referring to input views for training, and ‘-IO’ input views and network outputs for training. We compare our cell segmentation method performance associated with different learning strategies by (A) Precision, (B) Recall, (C) Intersection Over Union, (D) Dice Similarity Coefficient, (E) Correct Rate, and (F) Weighted Correct Rate. The marker † denotes the specific learning strategy used in S^4. The notations for *, **, and *** represent a p-value less than 0.05, 0.005, and 0.0005, respectively.

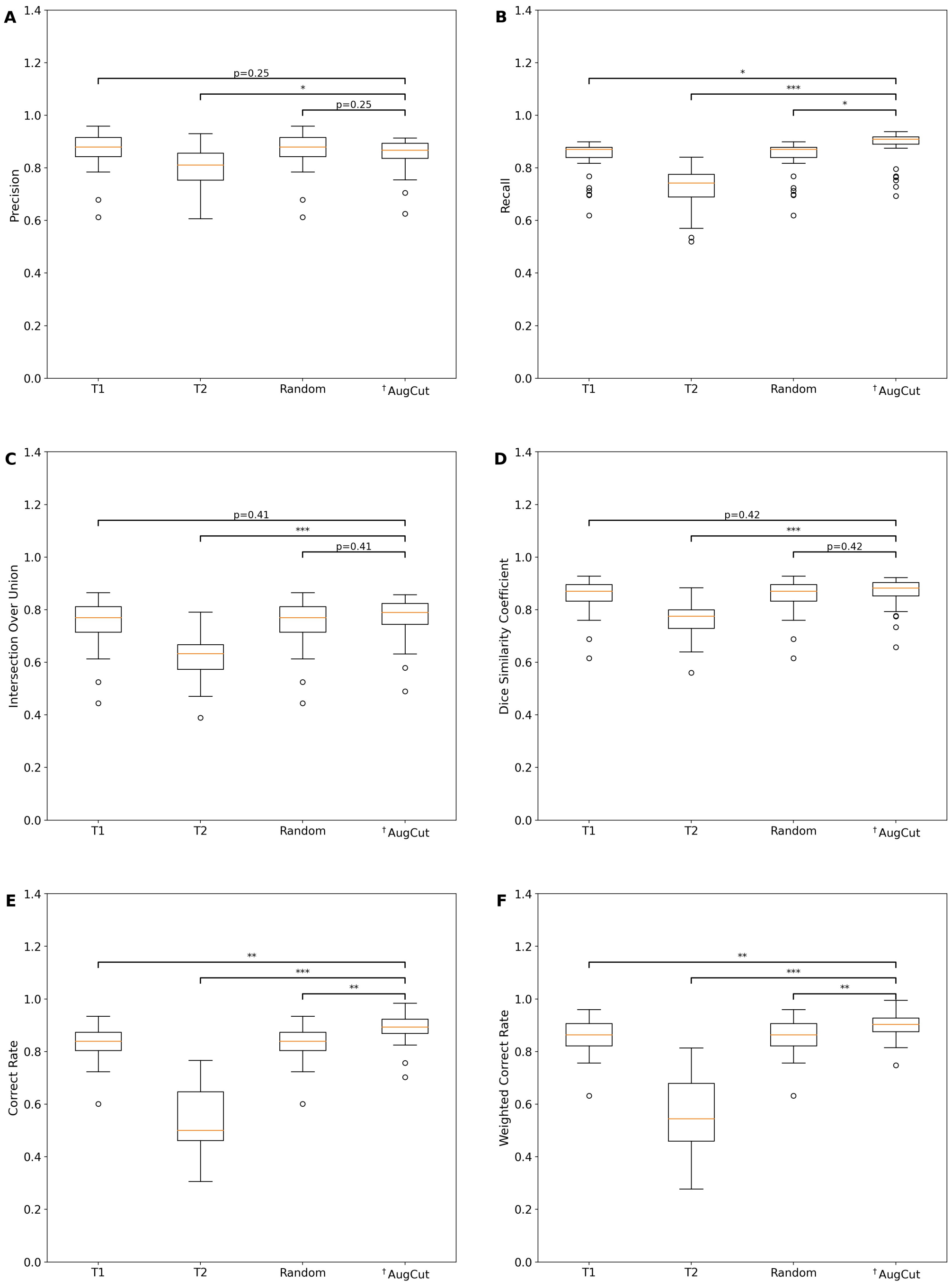

Fig. S4. Quantitative performance comparison across different image augmentation strategies by multiple evaluation metrics. We compare our cell segmentation method performance associated with different augmentation strategies by (A) Precision, (B) Recall, (C) Intersection Over Union, (D) Dice Similarity Coefficient, (E) Correct Rate, and (F) Weighted Correct Rate. The marker † denotes the augmentation strategy used in S^4. The notations for *, **, and *** represent a p-value less than 0.05, 0.005, and 0.0005, respectively.

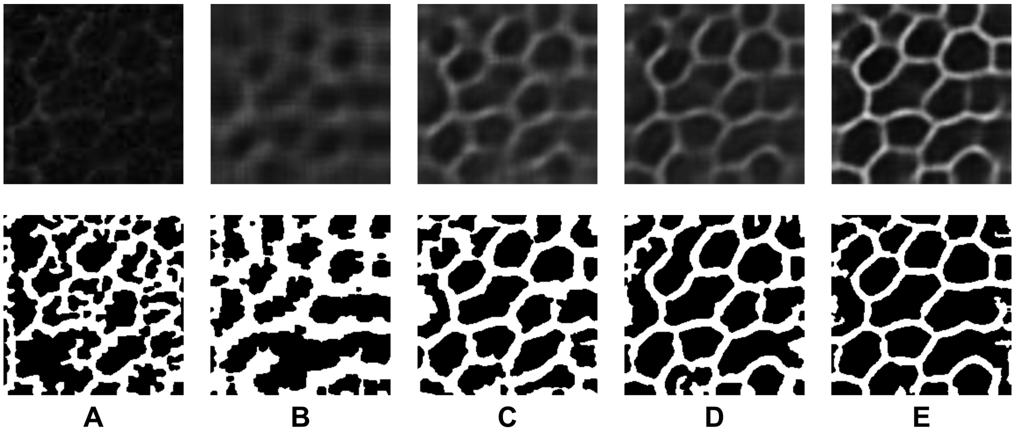

Fig. S5. The impact of pre-training epoch number on model outputs. A typical input image (top) to our model and its binary segmentation result from the morphological transformation process (bottom) are presented in (A). When the pre-training epoch number reaches (B) 20, (C) 40, (D) 60, and (E) 80, the associated outputs of the pre-training stage model (top) and the binary segmentation results from the morphological transformation process (bottom) are presented, respectively.

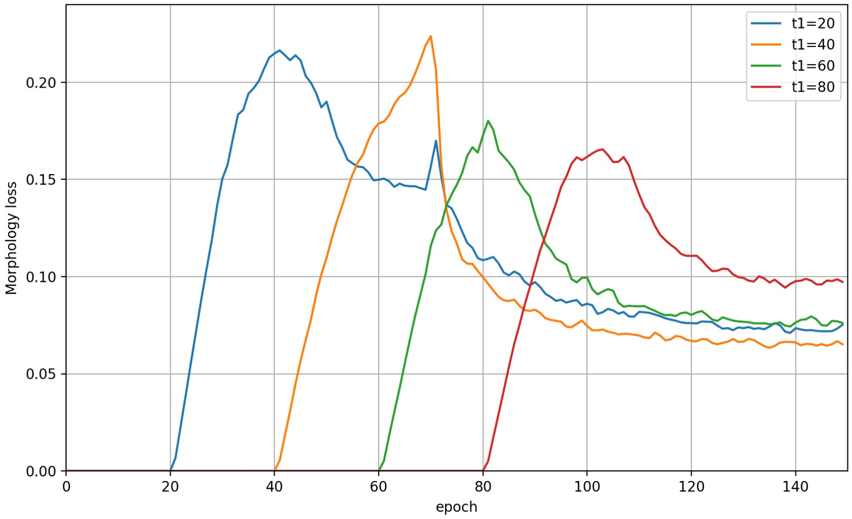

Fig. S6. Morphology loss curves associated with epoch number 20, 40, 60, and 80 for the pre-training stage, respectively. The resulting morphology loss curve associated with epoch number 40 converges to the lowest morphology loss, suggesting a proper epoch number for pre-training in our study.

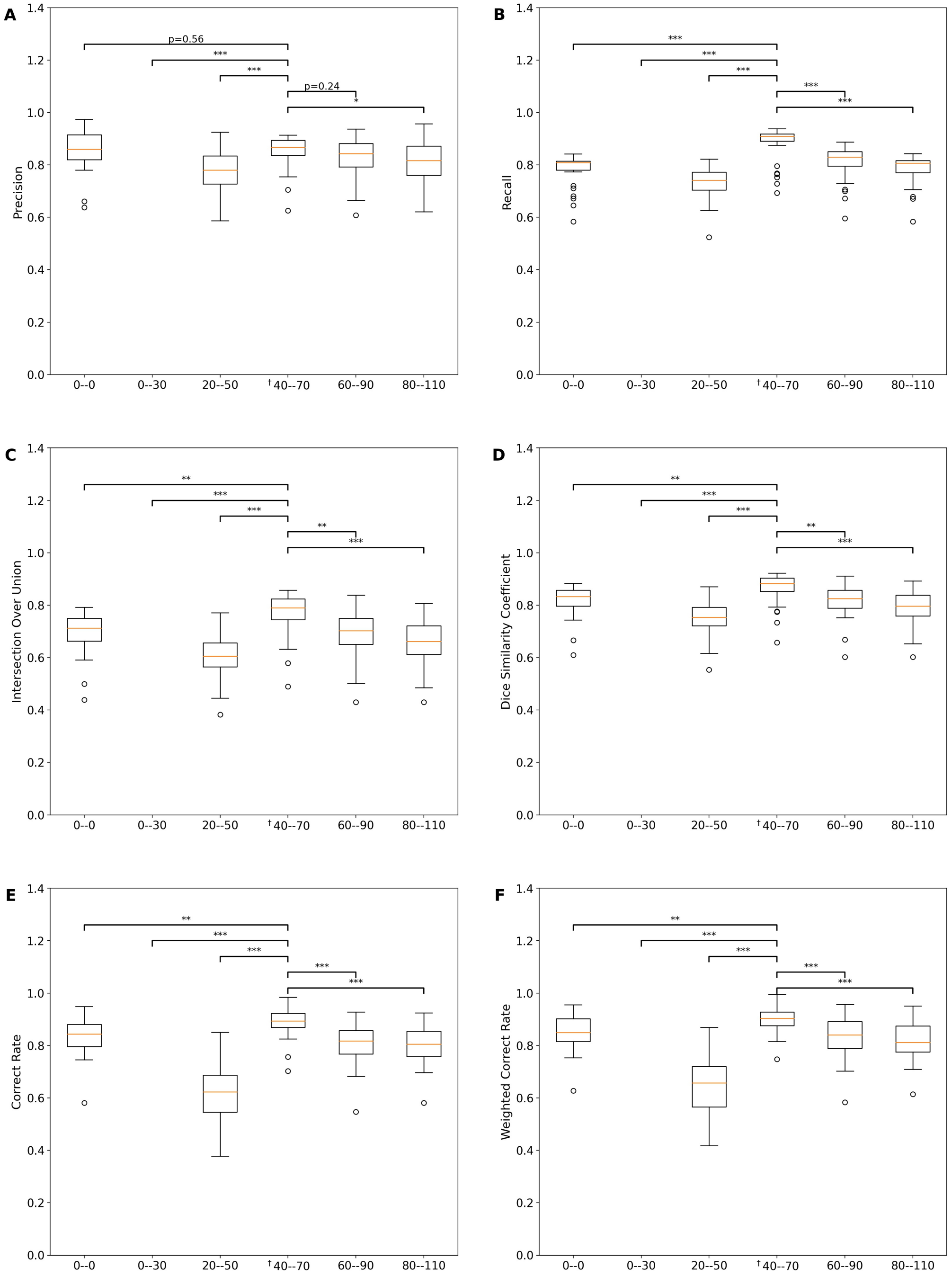

Fig. S7. Quantitative performance comparison across different training transition time cut-off values (in the format of '$t_1 - -t_2$') by multiple evaluation metrics. We denote t_1 and t_2 as the the starting and the ending transition time in the unit of epoch. Six pairs of transition time cutoff values are evaluated. We compare our cell segmentation method performance associated with different training transition time cutoff pairs by (A) Precision, (B) Recall, (C) Intersection Over Union, (D) Dice Similarity Coefficient, (E) Correct Rate, and (F) Weighted Correct Rate. The marker † denotes the specific time cutoff pair used in S^4. The notations for *, **, and *** represent a p-value less than 0.05, 0.005, and 0.0005, respectively.

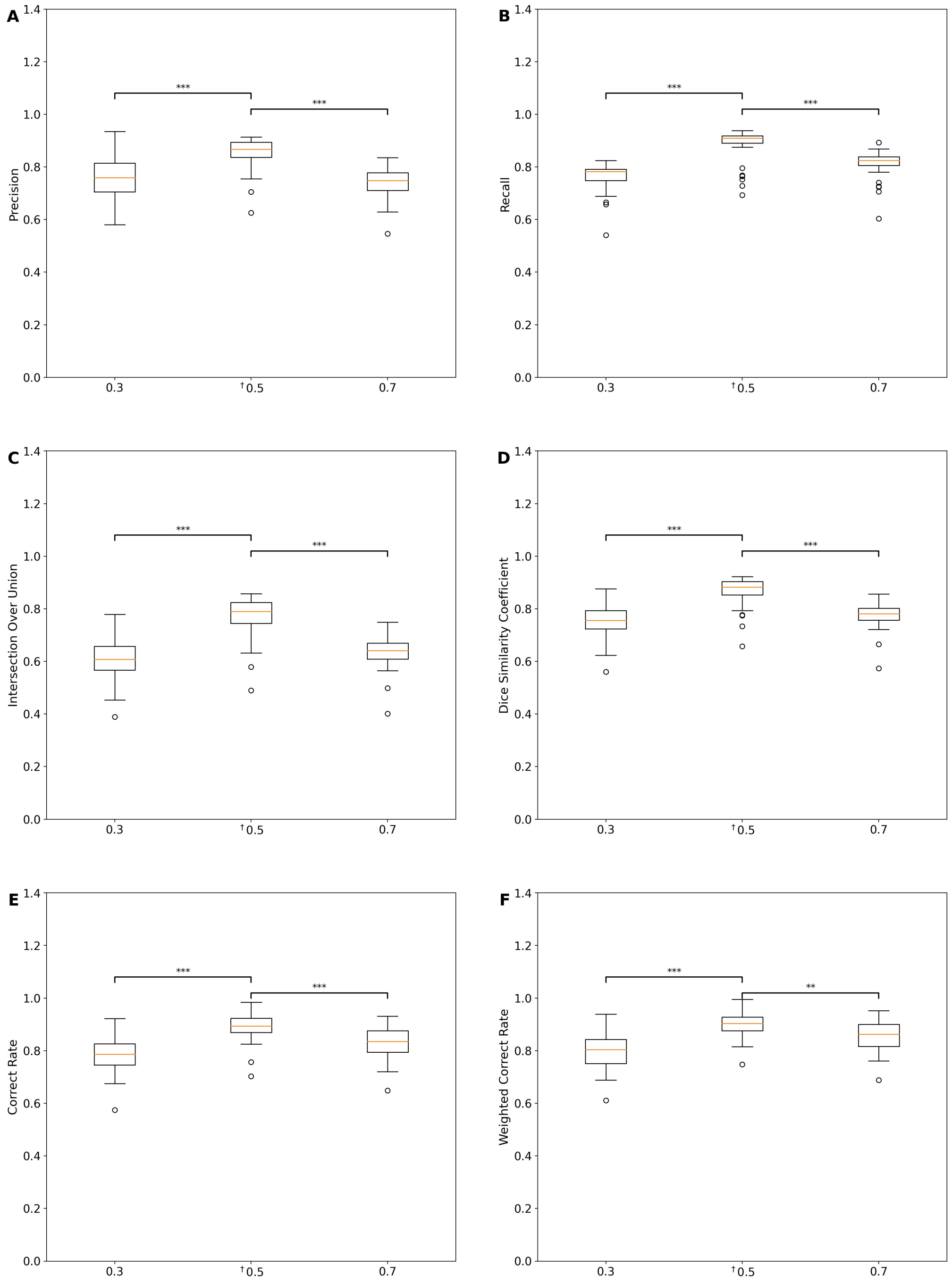

Fig. S8. Quantitative performance comparison across different stabilized values for weight factor λ_1. We compare our cell segmentation method performance associated with different stabilized values for weight factor λ_1 by (A) Precision, (B) Recall, (C) Intersection Over Union, (D) Dice Similarity Coefficient, (E) Correct Rate, and (F) Weighted Correct Rate. The marker † denotes the specific stabilized value used in S^4. The notations for *, **, and *** represent a p-value less than 0.05, 0.005, and 0.0005, respectively.
